# Clinicopathological, molecular and prognostic characteristics of cancer of unknown primary in China: An analysis of 1420 cases

**DOI:** 10.1002/cam4.4973

**Published:** 2022-07-13

**Authors:** Peng Qi, Yifeng Sun, Xin Liu, Sheng Wu, Yixin Wo, Qinghua Xu, Qifeng Wang, Xichun Hu, Xiaoyan Zhou

**Affiliations:** ^1^ Department of Pathology Fudan University Shanghai Cancer Center Shanghai China; ^2^ Department of Oncology, Shanghai Medical College Fudan University Shanghai China; ^3^ Institute of Pathology Fudan University Shanghai China; ^4^ The Cancer of Unknown Primary Group of Pathology Committee Chinese Research Hospital Association Shanghai China; ^5^ The Canhelp Genomics Research Center Canhelp Genomics Co., Ltd. Hangzhou China; ^6^ Department of Head & Neck Tumors and Neuroendocrine Tumors Fudan University Shanghai Cancer Center Shanghai China; ^7^ The Institute of Machine Learning and Systems Biology, College of Electronics and Information Engineering Tongji University Shanghai China; ^8^ Xuzhou Engineering Research Center of Medical Genetics and Transformation, Department of Genetics Xuzhou Medical University Xuzhou China; ^9^ Department of Medical Oncology Fudan University Shanghai Cancer Center Shanghai China

**Keywords:** cancer of unknown primary, clinicopathological characteristics, molecular feature, prognostic model, survival

## Abstract

**Background:**

Cancer of unknown primary (CUP) is defined the presence of metastatic disease without an identified primary site. An unidentifiable primary site of cancer creates significant challenges for treatment selection. We aimed to describe the clinicopathological, molecular, and prognostic characteristics of Chinese CUP patients.

**Methods:**

Patients with oncologist‐confirmed CUP were identified at Fudan University Shanghai Cancer Center from 2019 to 2020. Information on patient characteristics, tumor presentation, treatment, and outcome were retrospectively collected from the inpatient database and pathological consultation database for descriptive analysis. A multivariable logistic regression model was established to identify factors associated with patient prognosis.

**Results:**

A total of 1420 CUP patients were enrolled in this study. The baseline characteristics of the entire cohort included the following: median age (59 years old), female sex (45.8%), adenocarcinoma (47.7%), and poorly differentiated or undifferentiated tumors (92.1%). For the inpatient cohort, the most common sites where cancer spread included the lymph nodes (41.8%), bone (22.0%), liver (20.1%), and peritoneum/retroperitoneum (16.0%). A total of 77.4% and 58.2% of patients were treated with local therapy and systemic therapy, respectively. Four prognostic factors, including liver metastasis, peritoneal/retroperitoneal metastasis, number of metastatic sites (*N* ≥ 2), and systemic treatment, were independently associated with overall survival. Additionally, 24.8% (79/318) of patients received molecular testing, including PD‐L1, human papillomavirus, genetic variation, and 90‐gene expression tests for diagnosis or therapy selection.

**Conclusion:**

Cancer of unknown primary remains a difficult cancer to diagnose and manage. Our findings improve our understanding of Chinese CUP patient characteristics, leading to improved care and outcomes for CUP patients.

## Graphical abstract

In order to describe the clinicopathological, molecular and prognostic characteristics of Chinese CUP patients, we enrolled and analysised a total of 1420 CUP patients identified at Fudan University Shanghai Cancer Center from 2019 to 2020. Our findings improve our understanding of Chinese CUP patient characteristics, leading to improved care and outcomes for CUP patients.

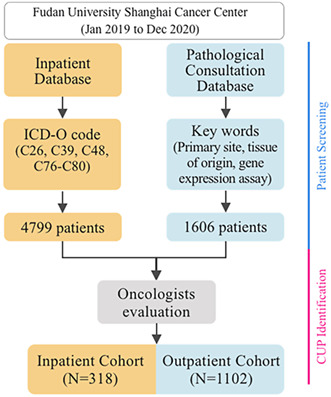

## 1 INTRODUCTION

Cancer of unknown primary (CUP) is a heterogeneous group of metastatic malignancies for which no primary site of the tumor is found using a standardized diagnostic approach.^1^ Increasingly, CUP is thought to include a distinct clinical subgroup of tumors with unpredictable but aggressive clinical behavior, including rapid growth, atypical metastatic spread, and multiple metastases that commonly spread to the lymph nodes, lung, bone, and liver.^2^ The unidentifiable primary site of cancer creates great challenges for the clinic, as the primary tumor site determines the treatment and overall prognosis of a patient.^3^ The diagnostic workup of CUP includes the use of some diagnostic technologies, such as modern medical imaging and endoscopy technologies, detailed histopathology, immunohistochemistry (IHC), and genetic testing.^4^ This may also be achieved by analyzing patient data to compare the incidence rates with those of known primary sites and tracking metastatic patterns of specific cancers.^5^

Historical studies have estimated that CUP accounts for 3%–5% of all new cancer cases; however, the exact incidence rate is difficult to determine for various objective reasons.^6^ In the current era, several studies have reported a decreasing trend for the occurrence of CUP with incidence rates of 1%–2%.^6^, ^7^, ^8^ The declining incidence of CUP is considered to be closely related to the development of diagnostic technologies, such as radiology, histopathology, and genetic testing, which are also reflected in improved completeness and validity of the cancer registration data.^9^ However, previous CUP epidemiological studies have focused on Sweden, Netherlands, Scotland, and United States. Relevant studies based on the Chinese population started relatively late and made slow progress, although these studies have received increasing attention in recent years.^5^, ^10^, ^11^, ^12^ In China, some CUP patients may have prolonged pathways to diagnosis and management and long hospital stays and may be discussed by multiple multidisciplinary teams (MDTs). CUP cases have been reported to include a disproportionately high number of adenocarcinomas and related cell types, and interestingly, some CUP subsets demonstrate long‐term survival after the administration of multiagent chemotherapy and other systemic treatments.^13^ Moreover, an epidemiological study of CUP is crucial to the screening and diagnosis of cancer as well as cancer etiology. Comparisons of incidence rates according to patients' homogeneous origins, analysis of causes of death, and comparison of the relative survival time are essential for the recognition of primary sites and tracking the metastatic process and thus provide even more insights into CUP etiology and therapy.^14^, ^15^

In the United Kingdom, the National Institute for Clinical Excellence (NICE) published guidelines in 2010 and recommended that every hospital with a Cancer Centre should establish a CUP team, including an oncologist, a palliative care physician, and a specialist nurse.^16^ Although the relevant guidelines have not been published in China, Fudan University Shanghai Cancer Center (FUSCC, Shanghai, China), which is one of the Chinese authoritative cancer specialist hospitals, has taken the lead in establishing the “Diagnosis and Treatment Center for Multiple or Unknown Primary Malignant Tumors”, aiming to supply advice on appropriate investigations and multidisciplinary review of cases and help reach a working diagnosis from which to coordinate anticancer‐specific treatments for CUP patients. In this study, we describe the epidemiological characteristics of CUP in FUSCC over 2 years. We aimed to complement CUP data routinely available from the Clinical Statistics Center by further investigating differences in CUP patients according to patient characteristics, tumor presentation, treatment, and outcome.

## 2 MATERIALS AND METHODS

### 2.1 Patient population

The present study was approved by the ethics committee of the FUSCC. The CUP patients were retrospectively collected from the FUSCC inpatient database and pathological consultation database from January 1, 2019 to December 31, 2020. The FUSCC inpatient database registers all newly diagnosed malignancies that have been treated in FUSCC. Detailed information on patient characteristics, tumor presentation, treatment, and vital status was recorded. Another database called the pathological consultation database registers all outpatients with information only about patient characteristics and tumor presentation.

For the inpatient database, the International Classification of Diseases for Oncology (ICD‐O) code was applied to screen CUP patients. Medical records were supplied by FUSCC for cancers registered from 2019 to 2020. FUSCC supplied anonymous records of all patients registered with the following ICD‐O classifications: C26 (Malignant neoplasm of other and ill‐defined digestive organs), C39 (Malignant neoplasm of other and ill‐defined sites in the respiratory system and intrathoracic organs), C48 (Malignant neoplasm of retroperitoneum and peritoneum), C76 (Malignant neoplasm of other and ill‐defined sites), C77 (secondary and unspecified malignant neoplasm of lymph nodes), C78 (secondary malignant neoplasm of respiratory and digestive organs), C79 (secondary malignant neoplasm of other sites), and C80 (malignant neoplasm without specification of the site). For the pathological consultation database, we used keywords including “primary site”, “tissue of origin”, and “gene expression assay” in the pathology reports to screen the possible CUP patients.

Finally, all initially screened patients were reviewed by FUSCC oncologists and pathologists to select CUP patients and subsequently divided into an inpatient cohort and an outpatient cohort. According to criteria defined in the 2015 European Society for Medical Oncology (ESMO) Clinical Practice Guideline, the inclusion criteria for the study were patients clinically diagnosed with CUP with histopathologically confirmed metastatic tumors without a detectable tumor tissue of origin after standard evaluation (medical history, physical examination, blood counts, chest‐abdomen‐pelvis computed tomography scans, and directed assessment of all symptomatic areas).^1^ The exclusion criteria were as follows: (a) Patients with no detailed medical history, physical examination, blood counts, and imaging results; (b) patients without microscopic confirmation (cytology, histopathology); and (c) patients with benign tumors, tumors with suspected primary tumor sites, or recurrent malignancies.

### 2.2 Statistical analysis

Descriptive statistics were used to summarize CUP patients' characteristics. For analyses involving metastatic sites, only those patients for whom the site of metastasis used to be registered were included. In addition, more than one metastasis of the same type was regarded as one, for example, lymph node metastases in inguinal and supraclavicular were considered as one metastatic site. Furthermore, we chose to report each of the metastatic sites, which resulted in a total number of metastatic locations that exceeded the total number of CUP patients given that each case can present diverse metastatic sites. Treatments were categorized as local therapy (radiotherapy or/and surgery) and systemic therapy (hormone treatment, chemotherapy, or/and immunotherapy).

The vital status of patients was evaluated on June 1, 2021, and overall survival (OS) was calculated based on all‐cause mortality. The survival curves were plotted using the Kaplan–Meier method, and the log‐rank test was used to assess differences among clinicopathological factors (sex, age group, histological types, tumor differentiation, metastatic sites, number of metastatic sites [≥2 vs. <2], local therapy, and systemic therapy). A univariable Cox logistic regression analysis was used to evaluate any association between clinicopathological factors and prognosis. A multivariate Cox proportional model was fit to assess the association between variables and OS, and the results were expressed as hazard ratios (HRs) and 95% confidence intervals (95% CI). To identify high‐risk CUP patients for disease‐specific death according to clinicopathological parameters, patients were classified into a high‐risk group and a low‐risk group based on dichotomization.

All statistical analyses were computed in R software (version 3.6.1) with packages including ggplot2 (version 3.3.0), survminer (version 0.4.9), and survival (version 3.2.11). The *p* value was computed two‐sided and considered statistically significant if *p* value < 0.05.

## 3 RESULTS

### 3.1 Patient characteristics

**FIGURE 1:**
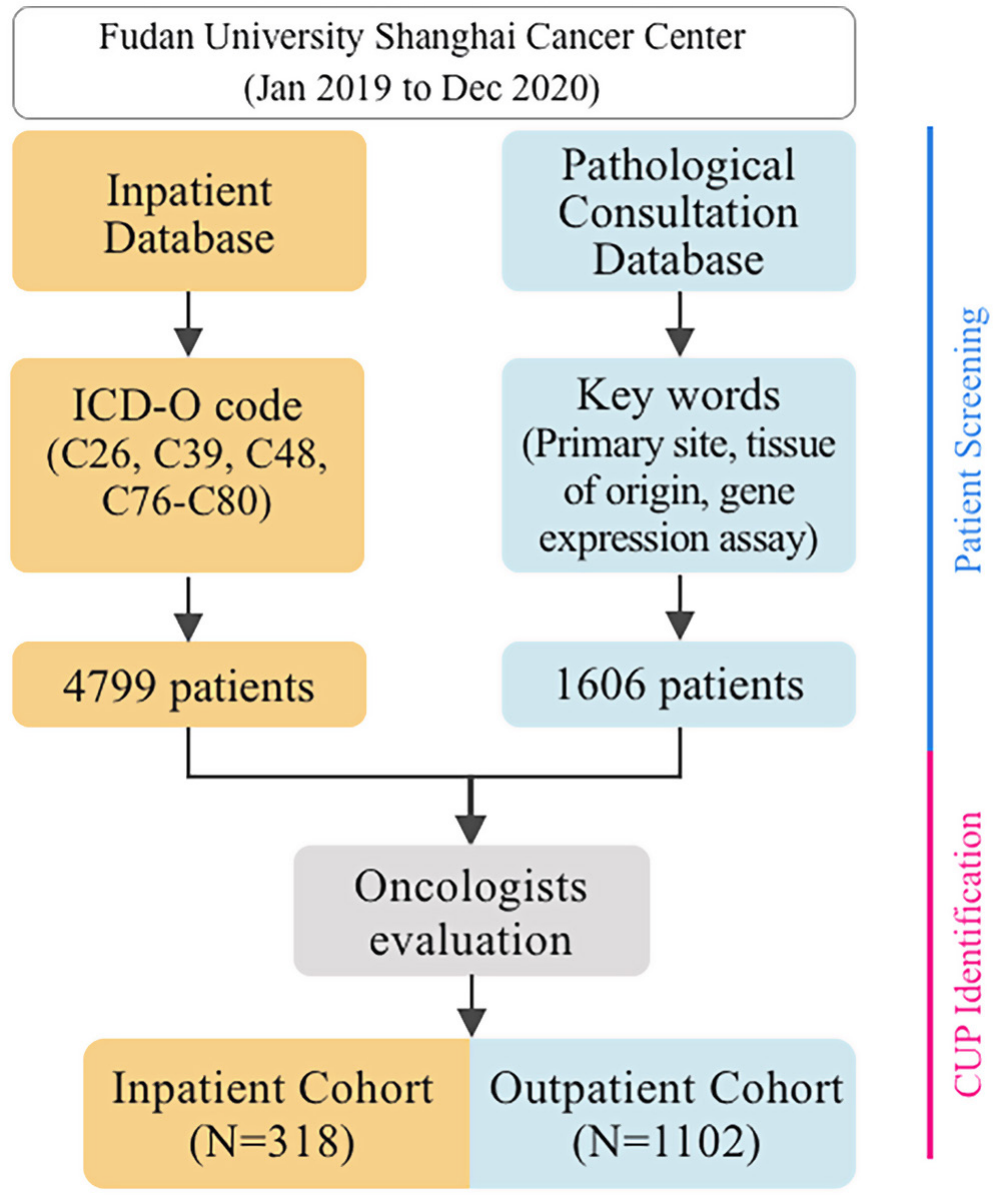
Patient screening and cancer of unknown primary identification flow

Between January 2019 and December 2020, a total of 1420 patients confirmed to have CUP by oncologists (PQ, XL, and QFW) were enrolled. Patient flow is shown in Figure 1. In general, CUP patients accounted for 0.82% (1420 of 173,581) of the total cancer diagnosis in FUSCC during the 2‐year period. The baseline characteristics of the outpatient cohort (*N* = 1102) and inpatient cohort (*N* = 318) are shown in Table 1. The baseline characteristics of the two cohorts were similar. The entire study population included 45.8% (*N* = 651) females and 54.2% (*N* = 769) males. The median age of all CUP patients was 59 years (range, 3–90). Among all the histological types, the majority subtype of CUP was adenocarcinoma (47.7%) followed by carcinoma (28.5%) and squamous cell carcinoma (17.0%). Regarding tumor differentiation status, 92.1% of CUP patients had poorly or undifferentiated tumors, whereas only 7.9% of specimens were well or moderately differentiated.

**TABLE 1:** Demographics characteristics of the outpatient cohort and the inpatient cohort

Characteristics	Outpatient cohort (*N* = 1102)		Inpatient cohort (*N* = 318)		Overall (*N* = 1420)	
	Number	%	Number	%	Number	%
Sex						
Female	514	46.6	137	43.1	651	45.8
Male	588	53.4	181	56.9	769	54.2
Age						
Median (range)	59	3–90	59	18–84	59	3–90
<50	270	24.5	70	22.0	340	23.9
50–59	285	25.9	92	28.9	377	26.6
60–69	370	33.6	111	34.9	481	33.9
≥70	177	16.0	45	14.2	222	15.6
Histological types						
Adenocarcinoma	522	47.4	155	48.7	677	47.7
Carcinoma	326	29.6	78	24.5	404	28.5
Squamous cell carcinoma	183	16.6	59	18.6	242	17.0
Neuroendocrine carcinoma	52	4.7	14	4.4	66	4.6
Melanoma	9	0.8	8	2.5	17	1.2
Sarcoma	10	0.9	4	1.3	14	1.0
Differentiation^a^						
Poorly/undifferentiated	670	92.8	168	89.4	838	92.1
Well‐moderately differentiated	52	7.2	20	10.6	72	7.9

^a^ The tumor differentiation of 510 specimens was undefined.

**FIGURE 2:**
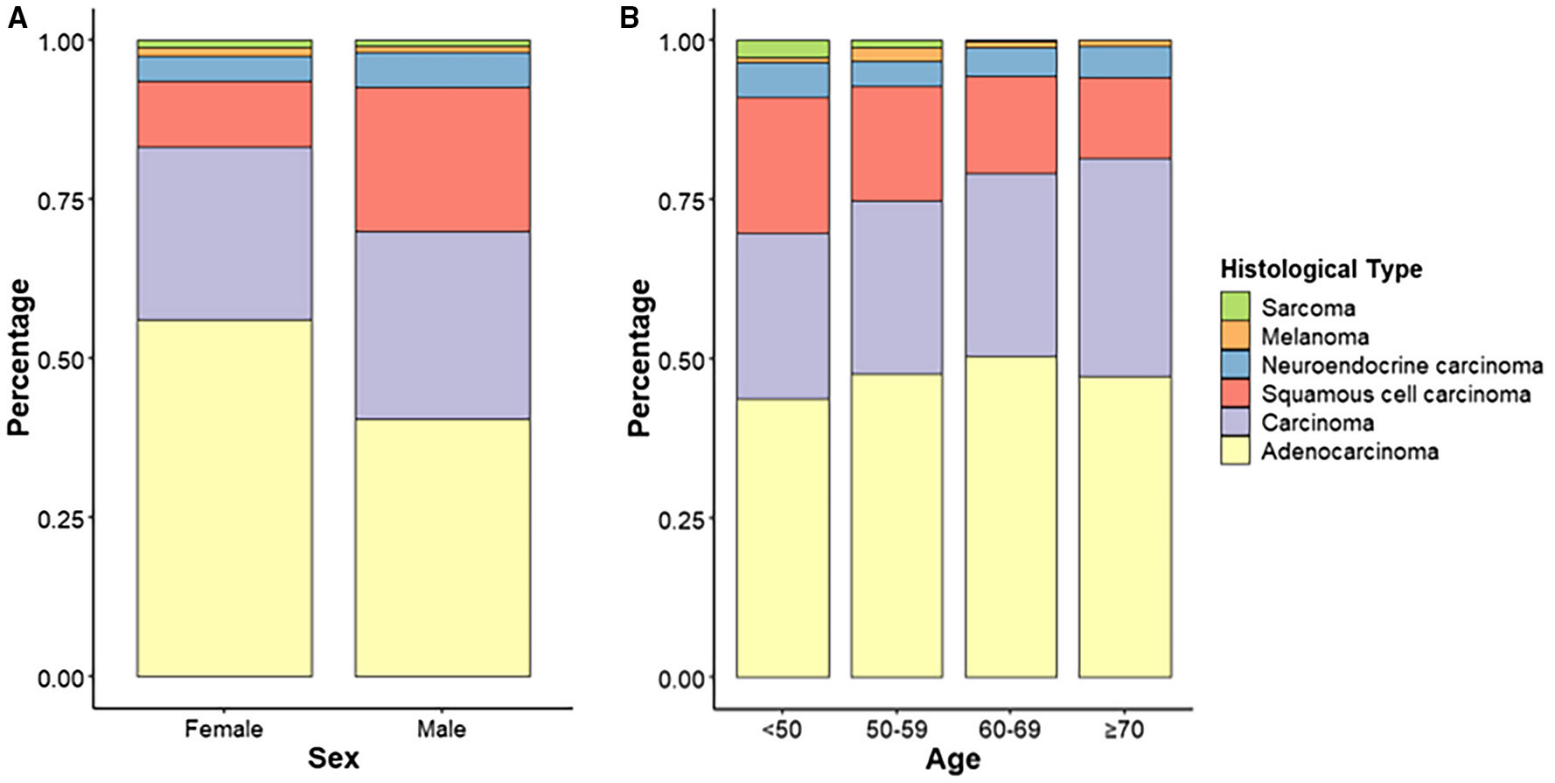
The proportion of histological types in cancer of unknown primary patients by (A) sex and (B) age

We further analyzed the correlation between age/gender and clinical characteristics. As shown in Figure 2A, the proportion of squamous cell carcinoma was higher in men compared with women, whereas the proportion of adenocarcinoma was lower than that in women. In addition, the proportion of squamous cell carcinoma decreased with age, whereas the percentage of carcinoma increased (Figure 2B).

In addition, for the inpatient cohort alone, detailed information on therapy and vital status was also recorded from the inpatient database (Table 2). Of 318 CUP patients, the most common sites where tumors spread include the lymph nodes (*N* = 133, 41.8%), bone (*N* = 70, 22.0%), liver (*N* = 64, 20.1%), peritoneum/retroperitoneum (*N* = 51, 16.0%), and lung (*N* = 27, 8.5%). Furthermore, we calculated the number of metastatic sites (involving the bone, liver, peritoneum/retroperitoneum, lung, and brain) for each CUP patient and found that 11.3% (*N* = 36) of cases had at least two metastatic sites. Regarding treatment, 246 CUP patients (77.4%) received local therapy, 185 CUP patients (58.2%) received systemic therapy, and only 6.3% (*N* = 20) of the patients did not receive any sort of treatment.

**TABLE 2:** Clinical characteristics of inpatient cohort

Characteristics	Inpatients cohort (*N* = 318)	
	Number	%
Metastatic sites		
Lymph nodes	133	41.8
Bone	70	22.0
Liver	64	20.1
Peritoneum/retroperitoneum	51	16.0
Lung	27	8.5
Brain	10	3.1
Number of metastatic site^a^		
*N* < 2	282	88.7
*N ≥ 2*	36	11.3
Local therapy^b^		
Yes	246	77.4
No	72	22.6
Systemic therapy^c^		
Yes	185	58.2
No	133	41.8

^a^ Limted to bone, liver, peritoneum/retroperitoneum, lung, and brain.

^b^ Local therapy including surgery, or/and radiotherapy.

^c^ Systemic therapy including chemotherapy, immunotherapy, or/and hormone therapy.

### 3.2 Molecular testing

Among the inpatient cohort, a total of 24.8% (79 of 318) of patients received molecular testing for diagnosis or therapy regimen selection utilities. More specifically, 26 cases underwent PD‐L1 IHC with a positive ratio of 38.5% (10 of 26). Twenty‐one patients underwent human papillomavirus (HPV) testing, including 19 high‐risk HPV types and five low‐risk HPV types. Interestingly, we found that the most common biopsy sites included the neck or neck lymph node (*N* = 13) and ovary/cervix (*N* = 3). Among 21 patients, 10 (52.4%) cases showed positive results, and all of them were HPV 16 type positive, indicating that these patients might potentially suffer from HPV‐related cancers, such as cervical cancer or oropharynx cancer. In addition, 38 cases underwent genetic variation testing, including multiplex genetic testing (*N* = 9) and single‐gene assays (such as *BRAF*, *RAS*, *EGFR*, *ALK*, etc.) (*N* = 29). The results showed that 16.6% (1 of 6) of patients had high microsatellite instability (MSI‐H), and 44.4% (4 of 9) of patients had potentially actionable genetic alterations.

**FIGURE 3:**
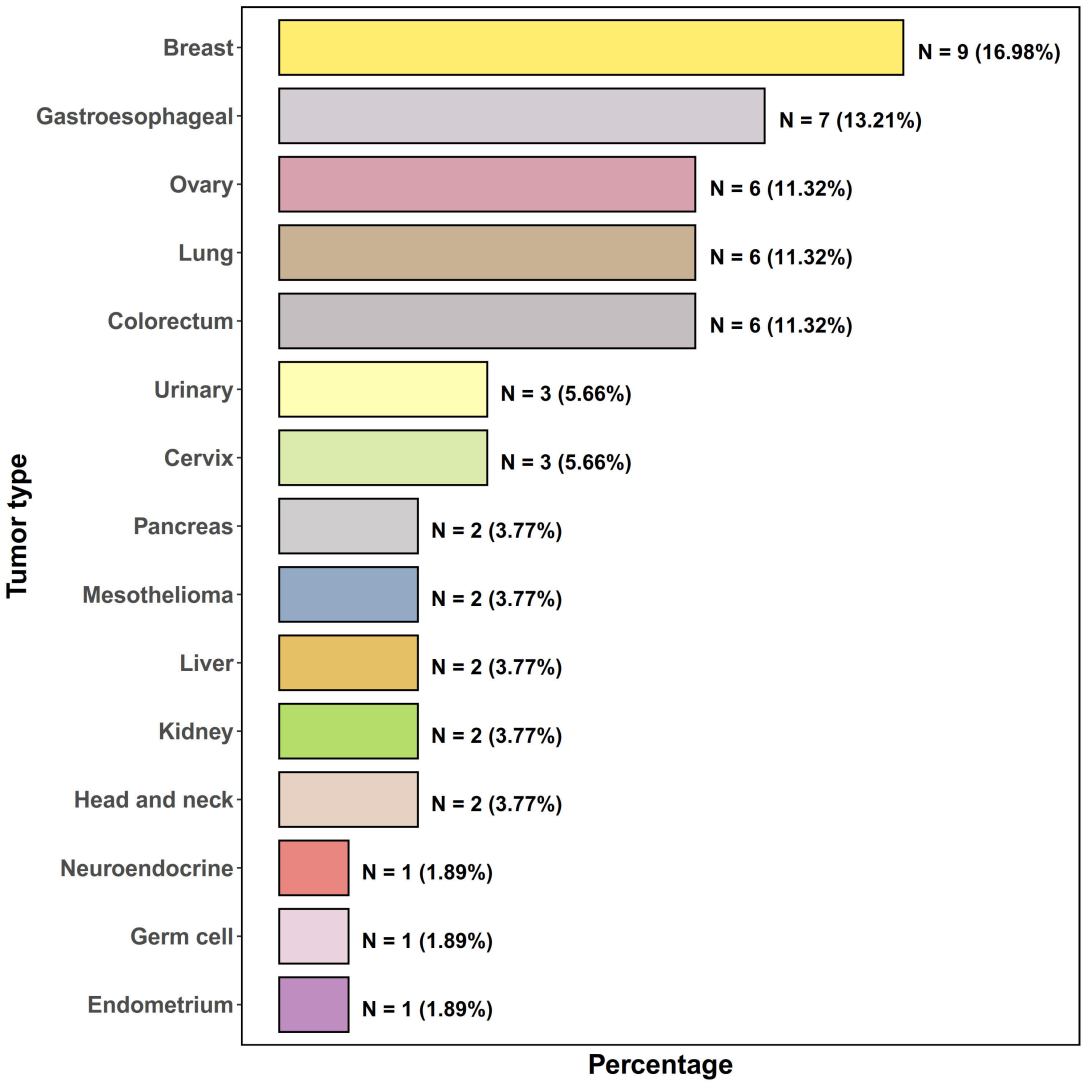
The distribution of tumor types predicted by the 90‐gene expression assay

More interestingly, a total of 58 patients underwent the 90‐gene expression assay (Canhelp Genomics Co., Ltd.) for primary site identification, and 91.4% (*N* = 53) of the patients had reportable results. Across all 53 successfully performed cases, the most common diagnoses were breast (*N* = 9, 17.0%), gastroesophageal (*N* = 7, 13.2%), ovary (*N* = 6, 11.3%), colorectum (*N* = 6, 11.3%), and lung (*N* = 6, 11.3%) (Figure 3). The method used for the 90‐gene expression assay is described in Appendix S1. Details of the 90‐gene expression assay results are presented in Table S1.

### 3.3 Survival analysis

**FIGURE 4:**
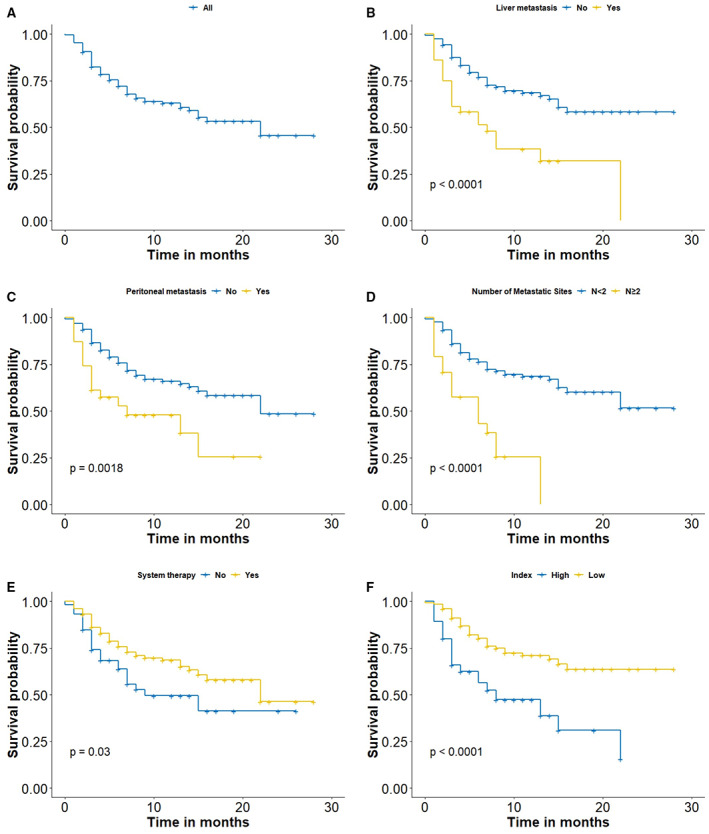
(A) The overall survival for the inpatient cohort. The overall survival of cancer of unknown primary patients by (B) liver metastasis, (C) peritoneal/retroperitoneal metastasis, (D) the number of metastatic organs, (E) systemic therapy, and (F) risk groups

We further analyzed the correlation between clinicopathological parameters and prognosis. A total of 190 CUP patients had survival information. The median survival time for these patients was 22 months with a range of 1–28 months (Figure 4A). Patients with liver metastasis (*p* < 0.0001, Figure 4B) or peritoneal/retroperitoneal (*p* = 0.0018, Figure 4C) metastasis showed a significantly poorer prognosis than those without metastasis at these two sites. These findings also indicated that more metastatic organs (*N* ≥ 2) led to a substantially increased risk of mortality (*p* < 0.0001, Figure 4D). Additionally, patients who underwent systemic therapy survived significantly longer than nonsystemic therapy patients (*p* = 0.03, Figure 4E).

### 3.4 Univariate and multivariable analyses

Among the inpatient cohort, the univariate and multivariable analyses of potential prognosticators are summarized in Table 3. Four factors, including liver metastasis, peritoneal/retroperitoneal metastasis, number of metastatic sites (*N* ≥ 2), and systemic treatment, were identified as independently associated with OS, and all were included in the multivariate Cox model. Patients suffering from liver or peritoneal/retroperitoneal metastasis, more metastatic sites (*N* ≥ 2) and those without systemic therapy demonstrated significantly inferior survival compared with those without peritoneal/retroperitoneal metastasis, fewer metastatic sites and those receiving systemic therapy, respectively (all *p* values < 0.05).

**TABLE 3:** Univariate and multivariate analysis of overall survival for the inpatient cohort (*N* = 318)

Characteristics	Univariate analysis			Multivariate analysis		
	Hazard ratio	95% CI	*p*‐value	Hazard ratio	95% CI	*p*‐value
Sex						
Female	Reference	—	—	—	—	—
Male	1.49	0.92–2.43	1.1E‐01	—	—	—
Age	1.02	0.99–1.04	1.3E‐01	—	—	—
Histological types						
Adenocarcinoma	Reference	—	—	—	—	—
Carcinoma	0.89	0.47–1.68	7.2E‐01	—	—	—
Melanoma	0.46	0.06–3.36	4.4E‐01	—	—	—
Neuroendocrine carcinoma	1.22	0.43–3.41	7.1E‐01	—	—	—
Sarcoma	0.83	0.11–6.16	8.6E‐01	—	—	—
Squamous cell carcinoma	0.72	0.38–1.35	3.0E‐01	—	—	—
Differentiation						
Poorly and Undifferentiated	Reference	—	—	—	—	—
Well or moderately	0.99	0.39–2.53	9.8E‐01	—	—	—
Lymph Nodes involvement						
No	Reference	—	—	—	—	—
Yes	0.79	0.49–1.27	3.3E‐01	—	—	—
Bone involvement						
No	Reference	—	—	—	—	—
Yes	1.35	0.77–2.37	2.9E‐01	—	—	—
Liver involvement						
No	Reference	—	—	—	—	—
Yes	2.77	1.66–4.61	9.1E‐05	2.09	1.12–3.9	2.0E‐02
Peritoneum/retroperitoneum involvement						
No	Reference	—	—	—	—	—
Yes	2.37	1.37–4.11	2.1E‐03	2.21	1.23–3.98	8.2E‐03
Lung involvement						
No	Reference	—	—	—	—	—
Yes	1.19	0.54–2.6	6.7E‐01	—	—	—
Brain involvement						
No	Reference	—	—	—	—	—
Yes	2.04	0.82–5.1	1.2E‐01	—	—	—
Number of metastatic Sites (N ≥ 2)						
*N* < 2	Reference	—	—	—	—	—
*N* ≥ 2	3.98	2.3–6.89	8.4E‐07	2.33	1.16–4.65	1.7E‐02
Local therapy						
No	Reference	—	—	—	—	—
Yes	0.7	0.42–1.17	1.7E‐01	—	—	—
Systemic therapy						
No	Reference	—	—	—	—	—
Yes	0.58	0.36–0.95	3.1E‐02	0.49	0.3–0.81	5.2E‐03

The HR of each variable for multivariate Cox regression analysis is shown in Table 3. In the multivariate analysis, patients who had more metastatic organs (*N* ≥ 2) had a significantly higher risk of mortality (*p* = 0.017), particularly for liver metastasis (*p* = 0.02) and peritoneal/retroperitoneal metastasis (*p* = 0.0082). It also indicated that patients who were treated with systemic therapy had a better prognosis (*p* = 0.0052). Between the systemic therapy and nonsystemic therapy subgroups, features including sex (*p* = 0.043), histological type (*p* = 0.030), lymph node involvement (*p* = 0.025), and bone involvement (*p* = 0.024) showed significant differences. The difference in patient characteristics between systemic and nonsystemic therapy is shown in Table S2. We further established a prognostic model with the basis of four significant prognostic variables. According to the median risk score, cases separated into low‐ and high‐risk groups showed a significant difference in survival (*p* < 0.0001, Figure 4F).

## 4 DISCUSSION

Our study explored the clinicopathological, molecular, and prognostic characteristics of CUP patients between 2019 and 2020 in FUSCC. Compared to the incidence rate of 3%–5% of cancer diagnoses in the historical series, the prevalence of CUP decreased to 0.82% in FUSCC, which is consistent with the latest epidemiological data of 1%–2%.^6^ This phenomenon can be attributed to the following reasons: Laboratory analysis, immunohistochemical diagnostic panels, molecular approaches, and advanced imaging techniques, such as computed tomography (CT) and positron emission tomography‐CT (PET‐CT) scans.^17^, ^18^, ^19^, ^20^ To date, no single strategy can currently be regarded as the gold standard for CUP diagnosis. In the clinic, a PET‐CT scan is typically used for primary site detection. One recent meta‐analysis of PET‐CT in 1942 patients from 20 centers found a primary tumor detection rate of 40.9% (39.0%–42.9%).^21^ In addition, due to the widespread use of gene expression assays (Tissue of Origin, CancerTYPE ID and the 90‐gene expression assay) for primary tumor identification with a sensitivity of 87%–90%, it is conceivable that the CUP incidence will continue to decrease in the future.^22^, ^23^, ^24^

In our study group, adenocarcinoma (47.7%) was the primary histological subtype, which is very similar to the proportion (48%) reported in the Netherlands CUP cohort.^12^ However, we report substantially increased percentages of carcinoma (28.5%) and squamous cell carcinoma (17%) in FUSCC compared with the Netherlands.^12^ This finding may also be due to coding methodology and ethnic differences. Interestingly, the incidence of the histopathological subsets differs between the age groups: older CUP patients have a higher incidence of carcinoma but a lower incidence of squamous cell carcinoma. These differences can be partly explained by the differences in the carcinogenesis of CUP between the age groups, which is still not completely understood.^25^ In addition, cancers in older patients may be affected by more environmental factors. An epidemiological analysis reported that adolescents and young adults had a higher incidence of squamous cell carcinoma than the general CUP population (29% vs. 10%), which is consistent with our results.^26^

Lymphatic metastasis is a relatively early process in distant metastasis and nodal metastasis for most solid tumors. Yilin et al. found that the sentinel lymph node is the first stop of lymphatic spreading of CUP cases, which helps track the primary tumor, especially for well‐differentiated tumors.^27^ In our study, as many as 41.8% of CUP patients suffered lymph node metastasis, demonstrating that the detection of tumor spread to lymph nodes helps track the primary site of CUP. Previously, researchers found that undifferentiated carcinoma patients had higher rates of advanced‐stage disease.^28^ In the present study, the grade of the tumor affects the ultimate identification of primary lesions, and a significant difference of 92.1% versus 7.9% for poorly or undifferentiated tumors and well or moderately differentiated tumors, respectively, was found. Thus, our results suggested that undifferentiated carcinoma seemed more likely to present early metastasis, making it more difficult to determine the tissue of origin for CUP patients.

In the initial pathological evaluation of CUP, morphology, and IHC, which are relatively cost‐efficient and nonburdensome for patients, contribute substantially to the targeted search for and identification of the primary tumor.^22^ However, IHC assessment helps pinpoint the probable site of origin in 10.8%–51% of CUP patients.^29^ Recently, gene expression profiling‐based assays have been quickly adopted in the field of CUP research.^22^, ^23^, ^24^ Gene expression analysis was characterized as the complement of the initial diagnostic workup to determine the tissue of origin in CUP patients, especially when IHC was inconclusive. In our study, 58 cases were assessed using the 90‐gene expression assay, and 91.4% of patients yielded reportable results. Currently, there is still no gold standard in the field of gene expression profiling tests, but molecular tests have the potential to significantly impact CUP patient management.^3^

A previous study reported that the CUP prognosis depends upon the locations of the primary tumors and the metastasis.^30^ Hemminki et al. also showed that site‐specific cancer deaths were related to metastatic sites.^31^ Although improved treatment and OS have been observed in some metastatic cancers,^32^ we found that CUP patients with liver metastasis or peritoneal/retroperitoneal metastasis had a poorer outcome. In addition, we observed a better prognosis for those patients who received systemic therapy compared with those who did not, which is consistent with previous studies.^33^ More interestingly, we also found a significantly increased percentage of women, adenocarcinoma, and lymph node involvement and a reduced percentage of bone involvement in patients who received systemic therapy, which is consistent with findings from the Netherland cohort.^12^ However, some results are difficult to interpret due to the wide range of patients and treatment options.^34^

Except for some subsets with favorable prognoses (15%–20%), treatment schemes for most CUP patients remain limited.^2^ Recently, the application of next‐generation sequencing (NGS) technologies to identify for potential therapeutic targets among CUP patients has begun to emerge.^3^, ^18^, ^35^ In our study, although only nine cases (2.8%) were performed using multiplex genetic testing, 44.4% (4 of 9) of patients had potentially actionable genetic alterations. Panels containing different genes may detect different mutations in the investigated cases. In the future, panel selection seems to affect the results of targetable mutations and subsequent treatments. A growing number of CUP patients may benefit from NGS technologies because 30%–85% of CUP patients harbor clinically relevant mutations.^36^ Precise and effective therapy may be available based on the molecular signature, and an improved prognosis is expected. Furthermore, the dynamic changes in the genomic and transcriptomic profiles of cancer can be monitored, and treatment schemes can be made accordingly.

This study still has several limitations. First, the unavailability of the full patient, treatment, and prognosis information of outpatients in this study makes it impossible to evaluate more complete epidemiological data. Second, this study is a single‐center study that only enrolled CUP patients treated over the past 2 years. In the future, we need to conduct a long‐term study in more centers across China to more comprehensively investigate the epidemiological situation of Chinese CUP patients. Third, the detailed information of treatment options and outcome after performing the gene expression assay or genetic tests is limited to assessing how the molecular test results could be translated into adapted therapy or improved prognosis. Herein, one prospective randomized study is currently being performed to further investigate the clinical utility of the 90‐gene expression assay for the identification of the tissue of origin and guiding treatment in CUP patients (Clinical Trials, NCT03278600).

## 5 CONCLUSION

Cancer of unknown primary remains a problematic cancer group to diagnose and manage. Our study assessed the true epidemiologic burden and clinicopathological characteristics of Chinese CUP patients. These data may lead to improved care and outcomes for CUP patients.

## AUTHOR CONTRIBUTIONS

Peng Qi, Yifeng Sun, Qinghua Xu, and Xiaoyan Zhou designed the study. Peng Qi, Xin Liu, and Qifeng Wang collected clinical and pathological information. Peng Qi, Yifeng Sun, Sheng Wu, and Yixin Wo analyzed all data. Peng Qi and Yifeng Sun wrote the initial manuscript draft. Qinghua Xu, Qifeng Wang, Xichun Hu, and Xiaoyan Zhou critically revised the manuscript and gave valuable insight to the study concept. All authors revised the manuscript. All authors read and approved the final manuscript.

## FUNDING INFORMATION

This work was partially supported by research funding from Innovation Program of Shanghai Science and Technology Committee grants 20Z11900300 (Xiaoyan Zhou), Innovation Group Project of Shanghai Municipal Health Commission grants 2019CXJQ03 (Xiaoyan Zhou), Shanghai Science and Technology Development Fund grants 19MC1911000 (Xiaoyan Zhou, Qifeng Wang, Peng Qi) and Shanghai Municipal Key Clinical Specialty grants shslczdzk01301 (Xiaoyan Zhou, Qifeng Wang, Peng Qi), the National Natural Science Foundation of China grants 81401963 (Qifeng Wang) and 81972728 (Qifeng Wang), Fudan University Shanghai Cancer Center grant YJYQ201603 (Qifeng Wang), Clinical Research Plan of SHDC grant SHDC2020CR3027B (Xichun Hu), and SHDC2020CR3046B (Xiaoyan Zhou).

## CONFLICT OF INTEREST

Author Yifeng Sun, Sheng Wu, Yixin Wo, Qinghua Xu were employed by the company Canhelp Genomics. The remaining authors declare that the research was conducted in the absence of any commercial or financial relationships that could be construed as a potential conflict of interest.

## ETHICS STATEMENT

Ethics approval and consent to participate for the study were obtained from the Clinical Research Ethics Committee of Fudan University Shanghai Cancer Center (Shanghai, China).

## CONSENT FOR PUBLICATION

Not applicable.

## Supporting information


Table S1.

Table S2.

Appendix S1.

## Data Availability

Data sharing is not applicable to this article as no datasets were generated or analyzed during the current study.
