# Supplementary material for: Clinicopathological, molecular and prognostic characteristics of cancer of unknown primary in China: An analysis of 1420 cases
Source: Cancer Med. 2022 Jul 13;12(2):1177–88. doi: 10.1002/cam4.4973 (PMC9883567; doi:10.1002/cam4.4973)
Supplement: Supplementary file 3 — Appendix S1. [file CAM4-12-1177-s003.docx]

**Supplementary Material**

**The method of the 90-gene expression assay**

The 90-gene expression assay (Canhelp Genomics Co., Ltd., Hangzhou, China) is a real-time PCR assay, which was developed for the classification of 21 common tumor types based on gene expression profiling. The 90-gene expression assay was carried out as previously described ^1^. In brief, the reverse transcription was performed on isolated total RNA. Next, the RT-PCR reaction was applied with a 7500 Real-Time PCR System (Applied Biosystems) to perform tumor-specific gene expression profiling. The internal control (IC) gene was used to assess the sample quality, while a weak RT-PCR signal (cycle threshold [Ct] value of the IC, greater than 38) was excluded. For each case, the 90-gene classifier analyzed the gene expression pattern of the 90 tumor-specific genes and generated similarity scores for each primary tumor type based on the degree of similarities of the test specimen to the gene expression database. The range of similarity scores was 0 (low similarity) to 100 (high similarity) for each tumor type, and the sum of similarity scores across 21 tumor types was 100. The tumor type with the highest similarity score was considered as the predicted tumor type by the 90-gene expression assay. In a retrospective cohort of 609 clinical samples, the gene expression assay demonstrated an overall accuracy of 90.4% for primary tumors and 89.2% for metastatic tumors. Furthermore, in a real-life cohort of 141 CUP patients, the gene expression assay was able to provide instructive predictions of primary tumors in 82.3% of patients (116/141) ^1^.

**Reference:**

1. Ye Q, Wang Q, Qi P, et al. Development and Clinical Validation of a 90-Gene Expression Assay for Identifying Tumor Tissue Origin. *The Journal of Molecular Diagnostics*. 2020;22(9):1139-1150.
